# Unified Nanotechnology Format: One Way to Store Them All

**DOI:** 10.3390/molecules27010063

**Published:** 2021-12-23

**Authors:** David Kuťák, Erik Poppleton, Haichao Miao, Petr Šulc, Ivan Barišić

**Affiliations:** 1 Business Unit Molecular Diagnostics, AIT Austrian Institute of Technology, 1210 Vienna, Austria; 2 Visualization Laboratory, Faculty of Informatics, Masaryk University, 60200 Brno, Czech Republic; 3 Center for Molecular Design and Biomimetics, The Biodesign Institute, School of Molecular Sciences, Arizona State University, Tempe, AZ 85281, USA; epopplet@asu.edu (E.P.); psulc@asu.edu (P.Š.); 4 Center for Applied Scientific Computing, Lawrence Livermore National Laboratory, Livermore, CA 94550, USA; miao1@llnl.gov

**Keywords:** DNA nanotechnology, file format, molecular file formats, computer-aided design, coarse-grained simulations, DNA origami, DNA-protein engineering, RNA nanotechnology

## Abstract

The domains of DNA and RNA nanotechnology are steadily gaining in popularity while proving their value with various successful results, including biosensing robots and drug delivery cages. Nowadays, the nanotechnology design pipeline usually relies on computer-based design (CAD) approaches to design and simulate the desired structure before the wet lab assembly. To aid with these tasks, various software tools exist and are often used in conjunction. However, their interoperability is hindered by a lack of a common file format that is fully descriptive of the many design paradigms. Therefore, in this paper, we propose a Unified Nanotechnology Format (UNF) designed specifically for the biomimetic nanotechnology field. UNF allows storage of both design and simulation data in a single file, including free-form and lattice-based DNA structures. By defining a logical and versatile format, we hope it will become a widely accepted and used file format for the nucleic acid nanotechnology community, facilitating the future work of researchers and software developers. Together with the format description and publicly available documentation, we provide a set of converters from existing file formats to simplify the transition. Finally, we present several use cases visualizing example structures stored in UNF, showcasing the various types of data UNF can handle.

## 1. Introduction

The steadily increasing popularity of the field of DNA nanotechnology has seen success in various domains, ranging from biomedical research [1], through drug delivery [2], up to the creation of nanorobots [3]. The basic premise of this field is that DNA molecules have predictable binding properties based on Watson–Crick base pairing of complementary sequences, allowing researchers to design strands that self-assemble into a vast range of nanoscale-sized structures. While these structures share the nanometer scale, they can significantly differ in terms of size, internal complexity, or the desired use case. Furthermore, despite the core Watson–Crick base-pairing rules being well-defined, in theory, the properties of nanostructures are also determined by a range of other factors in the real-world application, including charge interactions, kinetic traps, and knotting. These factors also partially depend on the chosen design paradigm. Amongst the most popular paradigms nowadays are multilayer DNA origami [4] and wireframe DNA origami [5,6]. Furthermore, in addition to DNA nanotechnology, there also exists an ever-growing field of RNA nanotechnology [7], sharing many of the same design concepts while leveraging the wider chemical capabilities of RNA, allowing for the incorporation of naturally occurring structural RNA motifs. Moreover, DNA nanostructures have started to be combined with proteins to create DNA-protein hybrid nanostructures [8]. These conjugates further increase the potential applications of nanotechnology. On the other hand, the design and assembly of such structures are correspondingly more complex.

As the nanostructures grow in complexity, it is desirable to utilize in-silico design tools to prevent expensive and time-consuming lab experiments. The typical modern DNA nanotechnology pipeline includes a basic design–simulation loop, allowing to design the structure using computer-aided design tools, followed by molecular dynamics simulations verifying the properties of the design. This pipeline often involves various software tools, such as Cadnano [9], Adenita [10], and oxDNA [11,12], focused on different subtasks. Ideally, it would be possible to directly use the outcomes of one tool as an input for another and then back again in an iterative manner, harnessing the strengths of the individual applications. However, the currently existing DNA nanotechnology software usually represents data in various formats, not offering sufficient support for such interoperability. This can be especially challenging in the case of collaborations between various research groups, where each prefers a different tool. This challenge can be partially overcome thanks to the file format converters, such as those provided by the TacoxDNA web server [13]. However, current export and conversion tools only convert structures from design-oriented file formats, such as Cadnano files, into simulation-ready formats, such as PDB and oxDNA. The opposite is currently not possible due to the loss of hierarchical information in the simulation formats. Furthermore, while existing formats have served the community well for the design of DNA structures, they generally lack support for emerging nanotechnology applications, such as RNA nanostructures and DNA-protein hybrids. As such, it is not surprising that various researchers, including us, lately called for a standard and sufficiently capable file format.

Therefore, in this paper, we propose a Unified Nanotechnology Format (UNF) designed specifically for the biomimetic nanotechnology domain, which uses DNA, RNA, and proteins as basic building blocks. The format aims to be usable in design and simulation applications, offering a verbose and explicit representation of the stored data to meet the needs of these areas. The in-depth documentation describing the format structure is available on a public repository (see Appendix A) to foster the inclusion in the existing and upcoming software tools. Together with the format description, we also provide a set of converters from existing file formats to simplify the transition to UNF. To showcase the capabilities of the UNF, various nanostructures stored in this file format are presented in this paper.

## 2. State of the Art

As soon as computers started to be used for the in-silico analysis and design of molecular structures, the need to store structural data in digital form naturally arose. There have been several distinct file formats developed over the years, storing various levels of information. For the description of atoms of a single molecule, MDL Molfile, structure-data file (SDF), and XYZ are often used [14,15]. All of them are text-based, with one line of text per atom. While XYZ omits storing explicit bonding information, MDL Molfile and SDF support this feature. In the area of proteins and other macromolecular structures, the most prevalent and de facto standard way of data storage is via the Protein Data Bank (PDB) format [16]. It paved the way for the standardization of biomolecular file formats, facilitating scientists’ work almost for half a century, thus having a significant influence on their work and achievements. Similar to the formats mentioned above, PDB also works in a text-based all-atom manner, storing one atom per line. The strength of PDB lies in its ability to define not only coordinates of molecules but also abstract molecular structure data, such as atom names, parent residue, multiple structure models, and crystallographic transformations. One disadvantage of the PDB format is its age, making it not well suited for the latest developments in the biomolecular domain, despite the numerous revisions over the years. For example, the fixed-column approach chosen by PDB limits the maximum number of atoms per model. For this reason, PDB is being replaced by its spiritual successor—the mmCIF (macromolecular Crystallographic Information) format [17,18]. It is also a textual format, but thanks to its dictionary-based approach, it is more flexible. Besides that, there are no limitations on the maximum number of atoms. On the other hand, when compared to PDB, it is less human-readable.

From the perspective of nanotechnology applications, PDB contains more details than necessary, but lacks a coarse-grained representation of structures. Most of the design tools for DNA nanotechnology work on a higher level of abstraction as they focus on nucleotide-level or helical segment representations, making the conversion to atomistic resolution difficult and often unnecessary. Current formats for storing coarse-grained molecular data are often closely coupled with particular molecular modeling software. For example, Moltemplate [19], a coarse-grained molecular builder for LAMMPS [20,21], uses a text-based file format, also known as LT (LAMMPS-Template format), closely resembling a source code. In HADDOCK [22], the all-atom to coarse-grained model conversion outputs a coarse-grained PDB file together with a file containing distance restraints, which is used when converting the coarse-grained data back to the fully atomistic model [23,24]. Finally, VMD [25] also allows its users to perform conversion to coarse-grained data, utilizing the MARTINI [26] CGC textual file format throughout the process [27].

Since the domain of DNA nanotechnology relies on in silico design and modeling of structures from scratch, the file formats must include additional data when compared to the general macromolecular ones. Moreover, due to existing design paradigms, it may be needed to capture some of their abstractions, not necessarily relevant to the structural data itself. As this domain can still be considered relatively young in comparison to other areas of molecular biology, the existing file formats reflect this by using more up-to-date technologies. Besides that, since it is often sufficient to work on the nucleotide level in nanotechnology, the existing file formats mostly omit atomistic details.

Probably the most widely used design file format is provided by the well-known DNA origami design tool Cadnano [9]. It stores data as a JSON (JavaScript Object Notation) file containing fields that describe the routing of single strands inside the DNA origami lattice. In more detail, Cadnano distinguishes between scaffold and staple strands, storing their data separately, but the information about the routing of individual strands is not explicitly stored and needs to be deduced. The disadvantage of Cadnano files is that they are limited to lattice-based design data. Furthermore, the nucleobase sequence needs to be stored in a separate CSV file. Some of the drawbacks of Cadnano’s file format are refined in the Scadnano [28] web-based DNA origami design application. It allows for storing the nucleotide sequences directly inside the file, includes chemical modifications, and is generally better structured. The JSON-based approach is also employed by the Tiamat design tool [29]. Compared to the Cadnano and scadnano file formats, Tiamat stores the world Cartesian coordinates for each duplex center line, or unpaired nucleobase, since it is a free-form design tool. Similarly, the Parabon inSēquio Design Studio application [30] also stores free-form data, using XML schema for their description. Finally, Adenita [10], an application for multiscale visualization and modeling of DNA nanostructures, employs a JSON-based file format to store the design files. An advantage of Adenita’s format is the ability to store both lattice-based data and free-form single strands. In some of the design tools, the used file format is closely coupled to the application framework in which they are implemented. For example, the file format of vHelix [6], an application focused on designing wireframe DNA origami, is based on Autodesk Maya ASCII files. Similarly, the file format of MagicDNA [31], a tool for multi-component DNA origami assembly, is based on Matlab files.

Among the approaches focused on nanostructure simulations and structure prediction, the oxDNA [12,32] file format stands out, having a robust suite of conversion tools into its format and a high degree of flexibility in its representation. OxDNA uses two text files—configuration and topology file—which store molecule positions/orientations and connectivity/sequence information, respectively. Furthermore, the recent extension of the oxDNA model, ANM-oxDNA, supports simulations and storage of DNA-protein or RNA-protein hybrid nanostructures [33].

A text-based file format is also employed by the 3D structure prediction software Cando [34], storing the necessary data for the reconstruction of single and double DNA strands, including the base-pairing information. The Structured Nucleic Acids Programming Interface SNUPI [35], a framework for computational analysis of DNA origami assemblies, employs several text-based formats, including XYZ, PDB, and Matlab data files. OxView [36], a freeform design tool and oxDNA configuration viewer, uses JSON-formatted files to store the scene data, which extends the data available in the oxDNA format by including information such as designed base pairs, alternate color schemes, labels, and grouped molecules.

**Figure 1:**
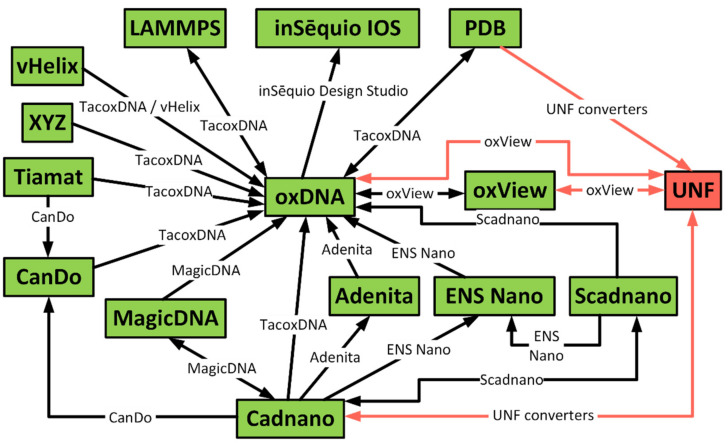
Visualization of the possible interconversions between the established nanotechnology formats and UNF. Graph nodes denote file formats while the edge labels mark the tools offering the conversion. oxDNA and Cadnano file formats are amongst the most important ones, while many of the conversions are realized via the TacoxDNA [13] converters and the oxView [36] application. The red-colored part shows the proposed Unified Nanotechnology Format together with the corresponding converters.

In total, the number of different file formats used in the nanotechnology domain is rather large, with most of the tools implementing their own instead of reusing existing ones (Figure 1). Thanks to the aforementioned TacoxDNA converter or built-in exporters, all the previously mentioned design formats can be converted into the oxDNA format for free-form editing with oxView. However, the situation is far from being ideal. OxView is a powerful tool for combining multiple designs and creating small free-form structures, but it lacks layers of abstraction such as virtual helices, domains, and scaffold routing, which allow other tools to design large and complex structures. Furthermore, the conversion to the oxDNA format results in the loss of design data which would be useful in the analysis and visualization of simulated structures.

Beyond the DNA itself, only Adenita and oxView allow the inclusion of RNA and protein structures within the design format. Moreover, no tool includes explicit representation of modified bases, small molecules, and other nanoparticles, such as gold nanoparticles and quantum dots, which are frequently found in DNA nanotechnology applications. Finally, many file formats are scarcely documented, if at all, making it difficult for software developers to understand their inner workings. Due to all of these reasons, we propose here a UNF, Unified Nanotechnology Format, described in the following section, which aims to provide an application-independent and well-documented solution to an omnipresent problem of nanotechnology data storage. By developing the UNF and converters into and out of the format, we hope to improve data sharing and interoperability among the tools and researchers involved in this field.

## 3. Format Description

In this chapter, the general structure of a UNF file, version 1.0, is introduced. The primary focus is on the concept as a whole, presenting the categories of data UNF handles. Therefore, most of the lower-level details are omitted in the text of the paper.

### 3.1. Overall Goal

As the name suggests, the proposed Unified Nanotechnology Format aims to provide a unified way of storing various nanotechnology structures, considering the specifics of existing design paradigms and the needs of simulation applications. In its current state proposed in the paper, the format definition is based on our survey of the available tools (described in Section 2), discussions with other experts, and our experience from developing in silico design tools for DNA and protein-based nanotechnology. Furthermore, there were several subgoals we aimed to achieve:

- Due to the popularity of the multilayer DNA origami technique, the format should be able to explicitly store designs of such lattice-constrained nanostructures. However, at the same time, it must also support the storing of the free-form DNA nanostructures, allowing for the description of arbitrarily shaped designs and simulation outcomes.
- The format should be viable for DNA-protein and RNA-protein nanotechnology engineering by storing coarse-grained representations of protein structures.
- For conversion from fully atomistic models to coarse-grained ones and vice versa, the format should have some way of referencing the original source data from crystallography, NMR, cryoEM, and all-atom simulation experiments.
- Related to the previous point, the format should support references to other types of molecules to allow the creation of more complex molecular scenes, possibly including all-atom structures together with coarse-grained ones.
- To facilitate the implementation of the format in various tools, it must be well defined, with a clear and properly explained terminology. Furthermore, the documentation of its structure should be easily available.
- The UNF file itself should be human-readable to allow for quick changes using a simple text editor in case of need. At the same time, it must be easy to process from the perspective of software developers.
- Ideally, the format should reuse well-defined concepts and terms from the existing DNA nanotechnology file formats and software applications to make the transition from other ways of data storage easier.
- Finally, due to the nature of the goal summarized at the beginning of this chapter, it is expected that the format will gradually evolve to meet the needs of its potential end-users. Therefore, it should be open for extension, making it possible to shape it in the future without a need for a complete rewrite.

Given all these subgoals, we realized that the file format used by Adenita might serve as a reasonable starting point and source of inspiration. Thus, we incorporated the advantages of this format and solved its limitations, extending it to go beyond its original possibilities. Furthermore, we also drew inspiration from file formats implemented by Cadnano and oxView as these are two well-known ones in the area of nanostructure design and molecular dynamics simulation, respectively.

### 3.2. UNF File Structure

**Figure 2:**
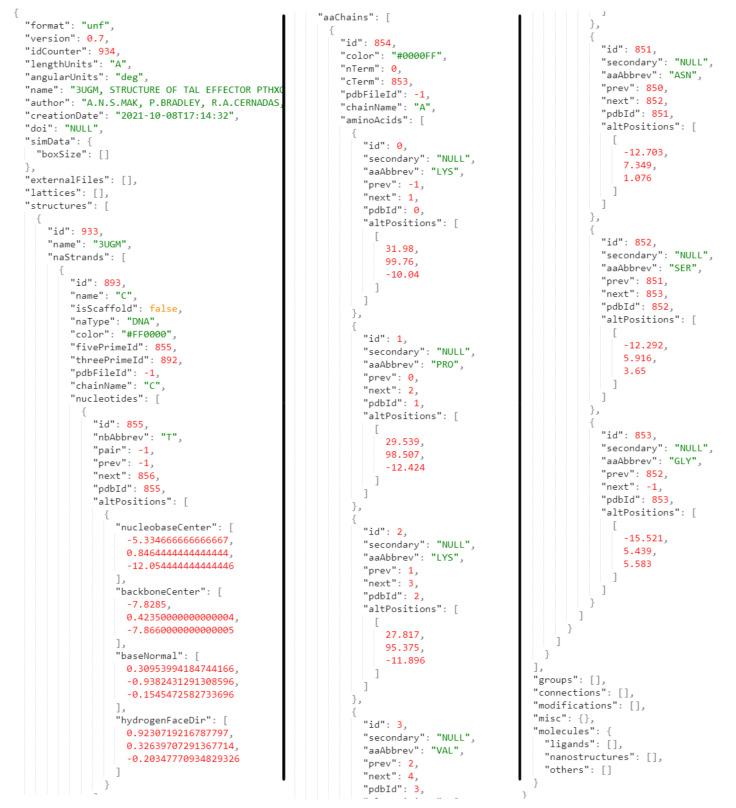
Excerpts from an example UNF file. Fields for identification of the file and stored structures are visible, together with parameters describing the location of nucleic and amino acids.

Similar to some of the existing nanotechnology formats, we decided to make the UNF JSON-based (see an excerpt from the example file in Figure 2). Apart from this approach being already well-explored in the domain, it has an advantage in both simplicity of file parsing and human readability. On the other hand, JSON files are not very space-efficient as they are rather verbose. However, given the focus on the complete definition of a coarse-grained structure, reasonable length is to be expected.

**Figure 3:**
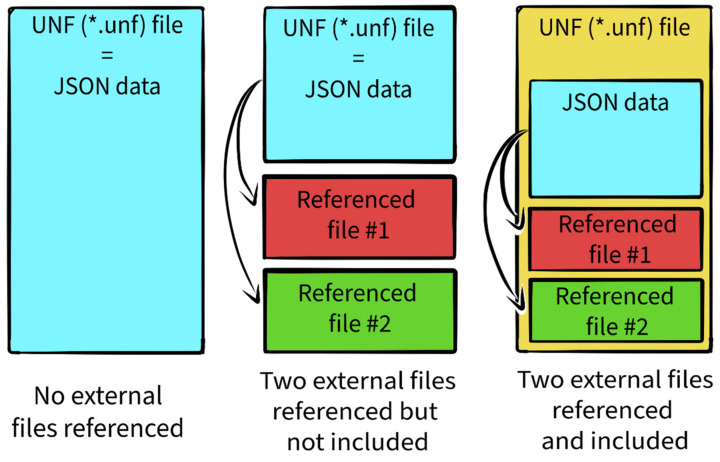
References to external files in UNF and their influence on the UNF file structure. The currently proposed solution allows for simple extraction of the JSON part if the remaining data are not needed in the given scenario, or are about to be processed later.

To enable the inclusion of fully atomistic molecules, UNF allows referencing external files (see Figure 3). These are primarily PDBs in UNF’s current state. Since a simple reference to an external file would mean that this file needs to be provided together with the UNF file to have complete data, UNF offers a way of including the content of other files directly in the UNF file, if needed. This makes it possible to distribute just one file containing all the necessary information. The inclusion of other files works by simply appending a specifically structured line of text at the end of the UNF file, followed by the contents of the appended file. The consequence of this approach is that the final UNF file is not always a clean JSON as it may contain other kinds of data appended to the end of the file. Furthermore, applications aiming to process these referenced files need to possess adequate parsers. However, extracting the JSON part out of the UNF file is a straightforward task. Besides that, since the JSON-stored data represent the main UNF content, referenced files serve mainly for specific use cases. They can be, therefore, omitted in some applications. For this reason, the content of referenced files is appended instead of being included directly in the JSON, as this reduces the memory and processing overhead posed on these kinds of applications.

### 3.3. Data Hierarchy

**Figure 4:**
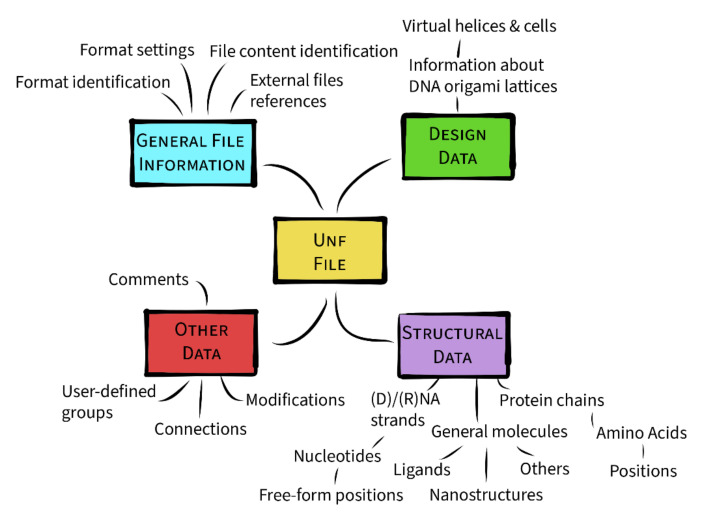
Categories of data stored by UNF. Each category carries a certain kind of information, with structural and design data being the most important content-wise as they define the structures.

The data represented by UNF can be split into four major categories, as visualized in Figure 4. While there is no one-to-one correspondence between these categories and the UNF fields, this distinction is helpful for the format description. Thus, in the following text, each of these categories will be introduced in more detail. To connect the textual description of the format with the UNF JSON attributes, terms emphasized in **bold** will refer to similarly-named JSON fields.

#### 3.3.1. General File Information

This group stores mainly data related to file identification and settings. They specify, for example, the **version** of UNF that is used and the units in which positions of objects are represented. Then, information about the file **name**, **author**, and **date of creation** are stored, as well as the **DOI** of relevant publications. Finally, references to **external files** are included. As for external files, except for the information mentioned in Section 3.2, UNF stores the MD5 **hash** of the file’s content for each referenced file. This ensures that the content of the given file is equal to its content at the moment of UNF creation.

#### 3.3.2. Design Data

Design-specific data are primarily represented by storing the information about DNA origami **lattices** to describe multilayer structures. For free-form structures, an explicit position of a nucleotide is stored, as described in the following section. UNF can encompass more than one lattice in a single file, making it well-suited for multi-component assembly. For each lattice, its **position** and **orientation** in space are saved, as well as its **type**, which can be either of the two arrangements permitted by Cadnano, square and honeycomb. Then, an array of its virtual helices is stored, where each virtual helix represents a possible location of a double helix. Virtual helices consist of **cells**, of which each references two arrays of nucleotides. One array corresponds to nucleotides from the single strand crossing the cell in the 5′ to 3′ direction, the other for a strand in the 3′ to 5′ direction. Furthermore, each cell possesses one of three **type**s of nucleotide information: Normal cells can reference up to one nucleotide in each of the arrays, signaling that this is a regular cell without any special properties. Deletion cells do not reference nucleotides as they denote that this cell should be skipped. Therefore, deletion cells allow the visual alignment of different lengths of double strands when designing structures. Finally, insertion cells allow for referencing more than one nucleotide. For the insertion of length *n*, the corresponding array contains *n* + 1 nucleotides. These concepts naturally correspond to the skips and loops in the Cadnano application, allowing the inclusion of these details in the UNF data. Overall, this hierarchy matches the semantic conventions of multilayer origamis used by Cadnano.

#### 3.3.3. Structural Data

At the core of structural data are coarse-grained **structures**, where each structure consists of an arbitrary number of **nucleic acid strands** and **amino acid chains**. All the necessary data are stored for both of these subunits, including the starting and ending amino and nucleic acids and a custom **color** for annotations. Nucleic acid strands also contain an attribute determining whether they are a scaffold or a staple strand. On top of that, they consist of **nucleotides**, storing the type of a nucleobase, neighboring nucleotides references, the paired nucleotide on the complementary strand, and the nucleotide’s position in space. For amino acid chains, similar information is saved, required for a successful reconstruction of a chain from the stored data. Since UNF operates on a coarse-grained level, it is essential to define how exactly the coarse-graining works. In this regard, UNF’s representation of nucleic and amino acid locations is based on models proposed by oxDNA, as it is verified both programmatically and experimentally [37,38]. In the case of protein structures, the **position** of an amino acid is represented by one vector corresponding to the location of its alpha carbon atom. Information about the side-chain orientation is not currently included as there are no tools that produce or read this information at the present time. For nucleic acids, four vectors are employed. Two of them describe the **center of mass** positions of the backbone and nucleobase. In this area, the UNF model deviates from the oxDNA representation, which stores only the center of mass of a whole nucleotide. We found out that extending the data model in this way allows for more flexibility and more straightforward visualization, particularly for structures converted from fully atomistic representations. The remaining two vectors uniquely describe the **normal of a nucleobase plane**, i.e., the base stacking direction and the **direction of a hydrogen bonding**. Since lattice-based design approaches do not explicitly store nucleotide locations, this situation needs to be captured by UNF as well. Therefore, a simple rule was proposed. If the nucleotide contains position records, then they take precedence over the lattice-based representation. Otherwise, the nucleotide is expected to be referenced by some lattice cell, and visualization tools can determine its position algorithmically.

UNF is also able to capture other kinds of **molecules**. For this purpose, the data model splits them into three groups: **ligands**, **nanostructures**, and **others**. Since ligands are usually small, they can be described as an explicit set of atoms and bonds. Nanostructures are currently identified by a name, reference to an external file, and position in space. The purpose of the nanostructures field is to store compounds such as gold nanoparticles. At the moment, the stored data are very general, as we aim to collect the needs of other experts over time to more precisely define what needs to be stored. Finally, the remaining field allows for the storage of arbitrary molecules referencing external PDB files. Thanks to this, UNF allows creating a molecular scene combining fully atomistic structures with coarse-grained ones.

#### 3.3.4. Other Data

The remaining category of data allows storing user-defined **groups**, **modifications**, **connections**, **comments**, and the size of the molecular **simulation box**. Groups are named as collections of IDs, allowing to reference various objects stored in a UNF for annotation purposes, clustering, and so on. Modifications enable storing of information about the nucleotides and amino acids that are chemically modified, together with the standard five and twenty- codes describing the type of modification. As for connections, they allow to explicitly define a particular interaction between several elements. Comments enable to store textual notes related to a particular element stored in the file. Finally, UNF also contains a field for **misc**ellaneous data. This is a general-purpose attribute where the individual applications can append arbitrary information while keeping the structure of the UNF file completely valid.

## 4. Converters from Existing Formats

Apart from proposing the format itself, we also provide a set of converters from some of the existing file formats to simplify the transition to UNF (Supplementary Material S1 and Appendix A). The Cadnano and PDB converters were implemented in the Python programming language and can be executed and parameterized via the command line, enabling their usage in automatized scenarios. The oxDNA/oxView converter is built into oxView, also creating the opportunity to edit UNF files as a result.

### 4.1. Cadnano ⇄ UNF Converter

**Figure 5:**
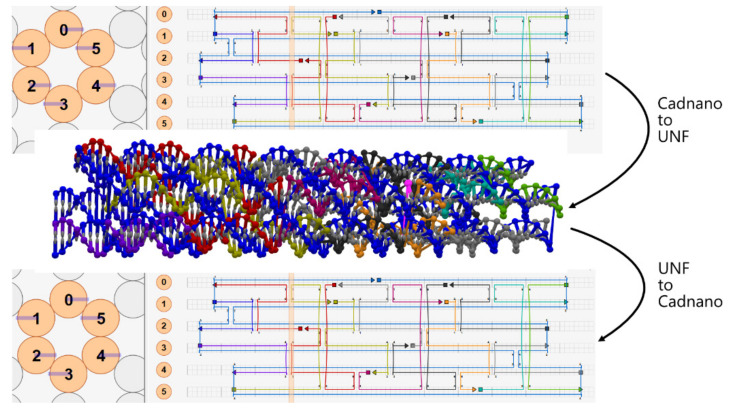
Process of converting files from Cadnano to UNF and then from UNF back to Cadnano, showcasing the supported interoperability between these two file formats. During both conversions, structural and color information remain fully preserved.

We provide both, Cadnano to UNF and UNF to Cadnano conversion (see Figure 5). The Cadnano to UNF converter allows converting multiple Cadnano files into one UNF file. For each input file, additional parameters, in the form of the lattice type and desired world space location of the lattice, are provided. Then, the converter extracts the design information from the Cadnano file and creates a new lattice record in UNF. Together with that, new nucleotides are created, referenced from the lattice **cells**, and assembled into **strands**. Cadnano’s loops and skips are converted to insertions and deletions, and the strands corresponding to these cells are modified in length accordingly. Circular scaffold strands are currently cut at a predefined location. While this is unnecessary for the UNF, it serves mainly as a convenience for the potential applications implementing the format. Since some Cadnano users utilize colors to annotate specific single strands, the staple strands coloring is also transformed to corresponding fields in UNF. In summary, for *n* input files, *n* records in **lattices** and **structures** UNF fields will be created.

In the case of UNF to Cadnano conversion, each **lattice** stored in UNF is converted to an individual Cadnano file. While this converter is still evolving, it already converts the most crucial features of the data. If the source UNF file contains free-form **structures** or additional **molecules**, they will be ignored during the conversion to Cadnano as this format does not support such data.

### 4.2. PDB → UNF Converter

This converter accepts a fully atomistic PDB or mmCIF file as an input and converts it to coarse-grained UNF representation, creating a new record in **structures**. Due to the types of data represented by UNF, only nucleic and amino acid chains together with ligands are processed; the rest of the file is ignored. It is expected that the PDB residues are listed in 5′ to 3′ direction, respectively, from the N-terminus to C-terminus.

An important part of the conversion process is the transformation of the atomistic locations to the reference frames of the UNF. This procedure directly corresponds to the way coarse-grained positional data are stored, as described in Section 3.3.3. For amino acids, the location of the alpha carbon is extracted. For nucleic acids, the process of conversion follows the one performed by the corresponding TacoxDNA converter. Therefore, vectors determining the orientation of a nucleobase are computed based on specific atoms and vectors between them. This procedure is detailed on the official UNF repository to ensure that all of the applications implementing UNF will behave consistently.

### 4.3. oxDNA/oxView ⇄ UNF

The UNF parser was written for the oxView visualization and editing application. It creates oxView system objects for each **structure** represented in the UNF. For structures containing **lattice** representations, positions in 3D space are determined based on the lattice corner coordinates, the position of the helix, and ideal B-form helix geometry. For structures containing **alternate positions**, positions of the nucleoside and backbone beads are set based on these coordinates. These systems can then be exported to the oxDNA or oxView formats using the built-in export tools. This provides UNF with an editing opportunity in an already available and widely used tool.

**Figure 6:**
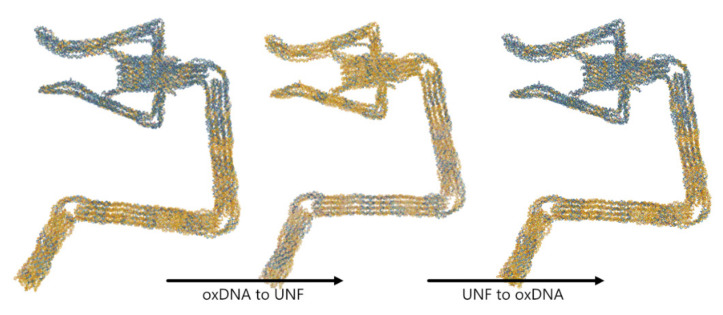
Structure of Robot arm with tweezer [31] converted from oxDNA to UNF and back using the oxView application.

OxView also contains an export option to the UNF format (see Figure 6). With the intended molecule loaded in the scene, the user can obtain a UNF representation by clicking the UNF export button in the “File” Table This will bring up a dialogue where the header information can be edited (it will be automatically filled from the most recently loaded UNF file in the scene if one exists). Currently, only the **structure** section of the UNF record is filled out as oxView does not have an internal representation of lattices. However, this is something we intend to support in the future. The export feature of oxView allows for editing of UNF structures as well as combining multiple files into a unified design file. As was described in Section 2—and visualized in Figure 1—the oxView plays, together with the tacoxDNA server, an important role for the interconversion of nanotechnology formats. Therefore, the option to import and export UNF and oxDNA files in oxView also enables users to store structures converted from many existing file formats in UNF, with the oxDNA file format being used as an intermediate step followed by the oxDNA to UNF conversion.

## 5. Use Cases

This chapter introduces example structures that UNF can process. They were created using the converters mentioned in Section 4, possibly combined with additional scripts and manual modifications. Some of these structures are available on the official repository of the format (Appendix A), together with documentation instructing how to reproduce the results from the source data. Since it would be challenging to imagine the contents of a UNF file purely from its textual representation, one of the tasks during the development of a new file format is to come up with a way how to visualize it. For this purpose, a web-based application called UNF Viewer was developed and made accessible on the official repository. Its sole purpose is to visualize the main contents of the UNF file, making it possible to verify if the structural data are correctly preserved during the conversion process or file modifications. Besides that, the oxView [34] application was also extended with UNF support, allowing for import, visualization, and editing of the stored structures. We hope that the extension of such a widely accepted tool with UNF support can significantly accelerate the inclusion of UNF in the work of other researchers.

### 5.1. Multi-Component Designs

**Figure 7:**
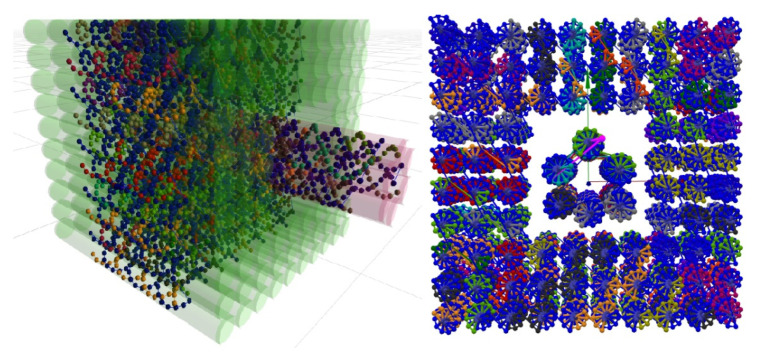
A hextube–cuboid structure stored in a single UNF file, visualized in UNF Viewer (**left**) and oxView (**right**), based on two different lattices. While the UNF Viewer also visualizes the empty lattice cells stored in UNF, oxView focuses purely on the structural data.

The first use case pertains to multi-component DNA origami designs. For example, in Cadnano, it is not possible to explicitly store nanostructure complexes consisting of more than one lattice. There are some workarounds, however, to achieve lattice structures where all lattices have the same arrangement. However, well-defined support for this kind of design is missing, despite this being a topic for many researchers [39,40,41,42]. In contrast, UNF was designed with a multi-component design in mind, naturally supporting such structures. Figure 7 shows a sample hextube–cuboid structure consisting of one honeycomb lattice design combined with a square lattice design. Data of both lattices, as well as their world locations, are stored in a single UNF file.

**Figure 8:**
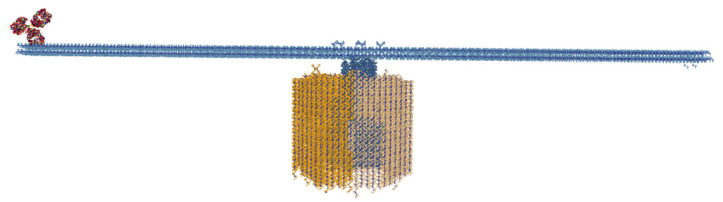
DNA origami rotor [42] together with an antibody (PDB: 1IGY [43]), visualized in oxView. An example of a multi-component DNA-protein hybrid design, which can be stored in UNF.

Another example of multi-component design is the DNA origami rotor structure, visualized in Figure 8, proposed by Ahmadi et al. [42]. This structure consists of four lattice-based components (three honeycombs, one square), which can be stored entirely in UNF. Moreover, using UNF we can also integrate the fully atomistic structure of a protein-based antibody to the given structure.

### 5.2. Multilayer DNA Origami Structures and All-Atom Molecules

**Figure 9:**
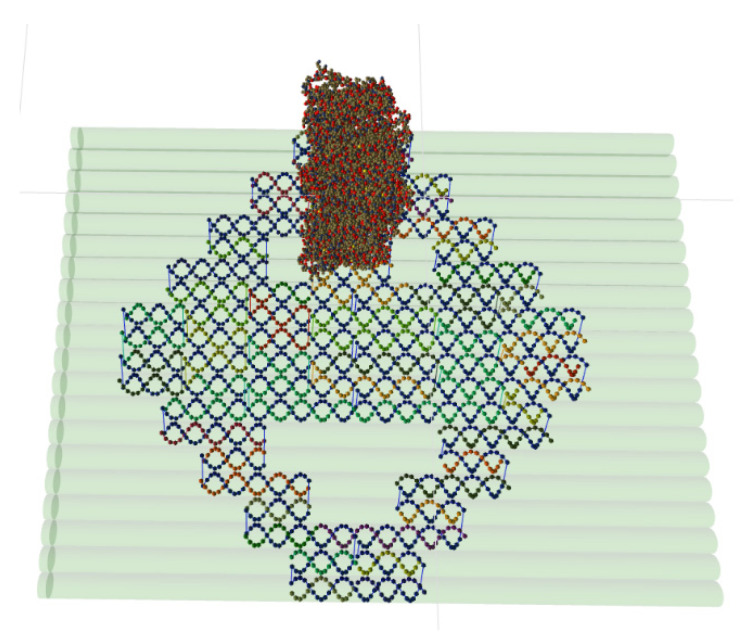
Smiley face DNA origami structure [44] combined with a protein (PDB: 6JI1 [45]), visualized in the UNF Viewer from the contents of a single UNF file.

As suggested by the DNA origami rotor structure in Figure 8, UNF can also store multilayer origami designs combined with fully atomistic structures. Another example of such a combination is visualized in Figure 9. To the best of our knowledge, this was not yet possible, in a straightforward way, with the publicly available formats. This allows for the creation of molecular sceneries combining different levels of detail. Thanks to that, coarse-grained structures can be modified in the corresponding design software while the information about the all-atom structure remains stored for molecular simulations performed after the design is finished.

### 5.3. Coarse-Grained DNA-Protein Hybrids

**Figure 10:**
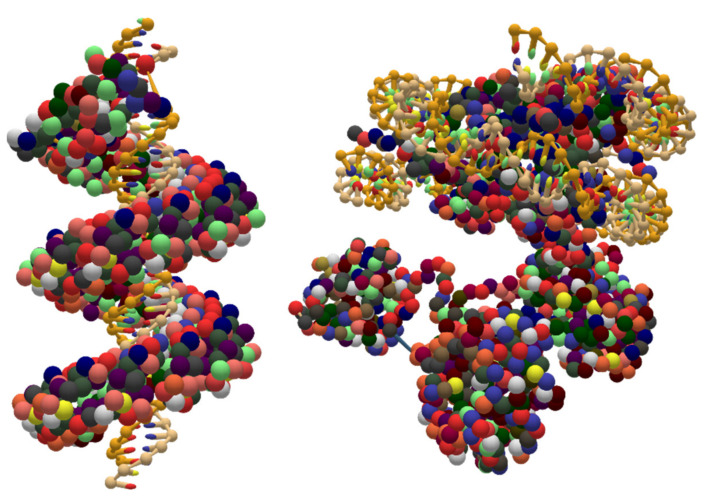
Two coarse-grained DNA-protein hybrids stored in UNF and visualized in oxView. The source data for the conversions to UNF were the PDBs 3UGM [46] (TAL protein) and 6KIX [47] (MLL1-NCP complex).

An important goal of UNF is the storage of coarse-grained structures, allowing to save arbitrary free-form designs. Two examples of such structures are visualized in Figure 10. Both of them result from PDB to UNF conversions using the converter described in the previous chapter. Apart from conversion from PDB, coarse-grained structures can also be designed from scratch in arbitrary tools offering this functionality. Moreover, they can be combined with lattice-based designs.

### 5.4. Coarse-Grained RNA Structures

**Figure 11:**
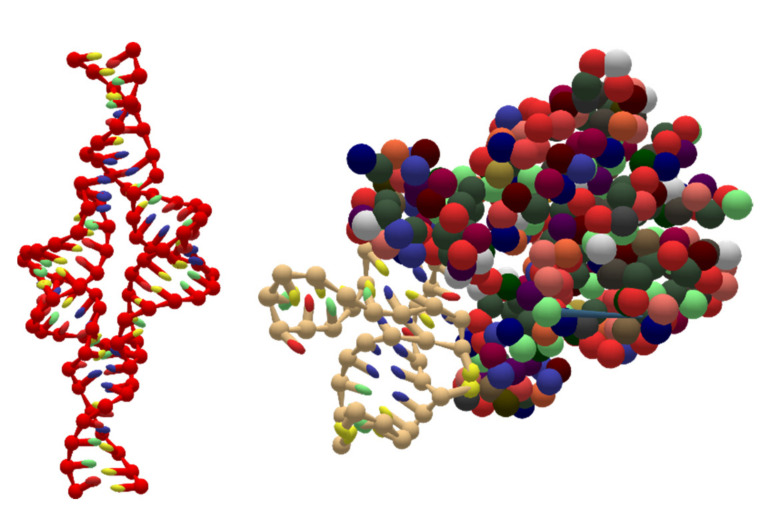
Two coarse-grained RNA structures stored in a UNF file and visualized in oxView. 2JYH [48] RNA is shown on the left, while 6SY6 [49] structure, which contains both RNA and protein, is on the right.

Due to the rise of RNA nanotechnology, UNF also supports coarse-grained RNA structures and their combination with proteins, as shown in Figure 11. As in the case of DNA structures, the PDB to UNF converter enables the conversion of the atomistic data to a coarse-grained UNF representation while correctly detecting the type of nucleic acid to label the given strand as RNA.

## 6. Conclusions

The Unified Nanotechnology Format—UNF, proposed in this paper, aims to provide a single way of data storage in the domains of DNA and RNA nanotechnology. It allows for the combination of different types of structural data, including multilayer DNA origami structures, free-form structures, coarse-grained amino acid chains, and fully atomistic molecules. By doing so, it should be suitable for both computer-aided design and molecular simulation areas with respect to the nanotechnology domain. The format description is publicly available, making it accessible to anyone who aims to implement it in their software framework. Moreover, since the format is sufficiently capable, well-structured, and thoroughly documented, such implementation should not pose a major challenge. Together with the format definition, a set of converters from other existing file formats is provided to facilitate the transition to UNF. To allow for visualization of the UNF-stored data, a UNF Viewer (see Section 5) application was developed. Moreover, the web-based oxView [34] application was extended to support UNF. By executing all of these steps, we believe that we can increase the interoperability and compatibility between individual software tools by providing a single means of data storage and exchange. Since the lack of a common file format for the nanotechnology domain has been lately discussed among various research groups, UNF presents a first major step in this direction. While we support most of the structures represented by well-established formats, there still exists specialized data, which UNF cannot fully represent. For example, while UNF can store wireframe DNA origami structures using the free-form nucleotide coordinates, it cannot meaningfully represent the polygonal mesh files used as source design data. Similarly, in the case of RNA structures, UNF can describe their spatial properties but does not offer an explicit way of storing the RNA origami blueprints used for the design. However, there are fields that can be utilized for this purpose without breaking the format compatibility. In any case, we realize that there might be novel design paradigms or discoveries in the future, posing additional requirements on the UNF in order to preserve its status of universally applicable format. For this reason, one of the important parts of the UNF ecosystem is a well-defined versioning system, allowing for tracing the history of format changes, as well as for quick identification of whether the given version of the format is compatible with another one.

With these points in mind, the format was designed to be open for further improvements. Therefore, we aim to collect feedback and opinions of additional experts to make the UNF suit their needs. Furthermore, we would like to extend some of the file format fields to make them more focused on a particular type of data. We will encourage and offer help to nucleic acid nanotechnology tool developers to support the export and loading of UNF format, as well as the development of interconversion tools between respective design formats to make sharing designs between research groups easier. In the end, we believe that UNF has the potential to replace the existing file formats where possible, as well as to open doors to new discoveries and interoperability between the individual software tools.

## Data Availability

The data presented in this study are available on the following repositories: https://github.com/barisicgroup/unf and https://github.com/sulcgroup/oxdna-viewer (accessed on 15 November 2021).

## Supplementary Materials

The following supporting information can be downloaded, ZIP archive S1: the contents of the UNF release 1.0.0.

## Sample Availability

Samples of the compounds are not available from the authors.

## Appendix A

- The official UNF repository is accessible via the following link: https://github.com/barisicgroup/unf (accessed on 15 November 2021).
- The repository of the oxView application is available using this link: https://github.com/sulcgroup/oxdna-viewer (accessed on 15 November 2021).
