# Supplementary material for: Unified Nanotechnology Format: One Way to Store Them All
Source: Molecules. 2021 Dec 23;27(1):63. doi: 10.3390/molecules27010063 (PMC8746876; doi:10.3390/molecules27010063)
Supplement: Supplementary file 1 [file molecules-27-00063-s001.zip › unf-1.0.0_finalized/unf-web-viewer/index.html]

UNF Web Viewer


# UNF Web Viewer

Simple web viewer visualizing the data stored in the Unified Nanotechnology Format (UNF) file.

Load UNF file:


File info:

No file loaded

Parsed file content:

UNF JSON structure visualizer:

(resize this window as needed)

+ Expand JSON - Collapse JSON
