# Supplementary figures and images for: Unified Nanotechnology Format: One Way to Store Them All
Source: Molecules. 2021 Dec 23;27(1):63. doi: 10.3390/molecules27010063 (PMC8746876; doi:10.3390/molecules27010063)

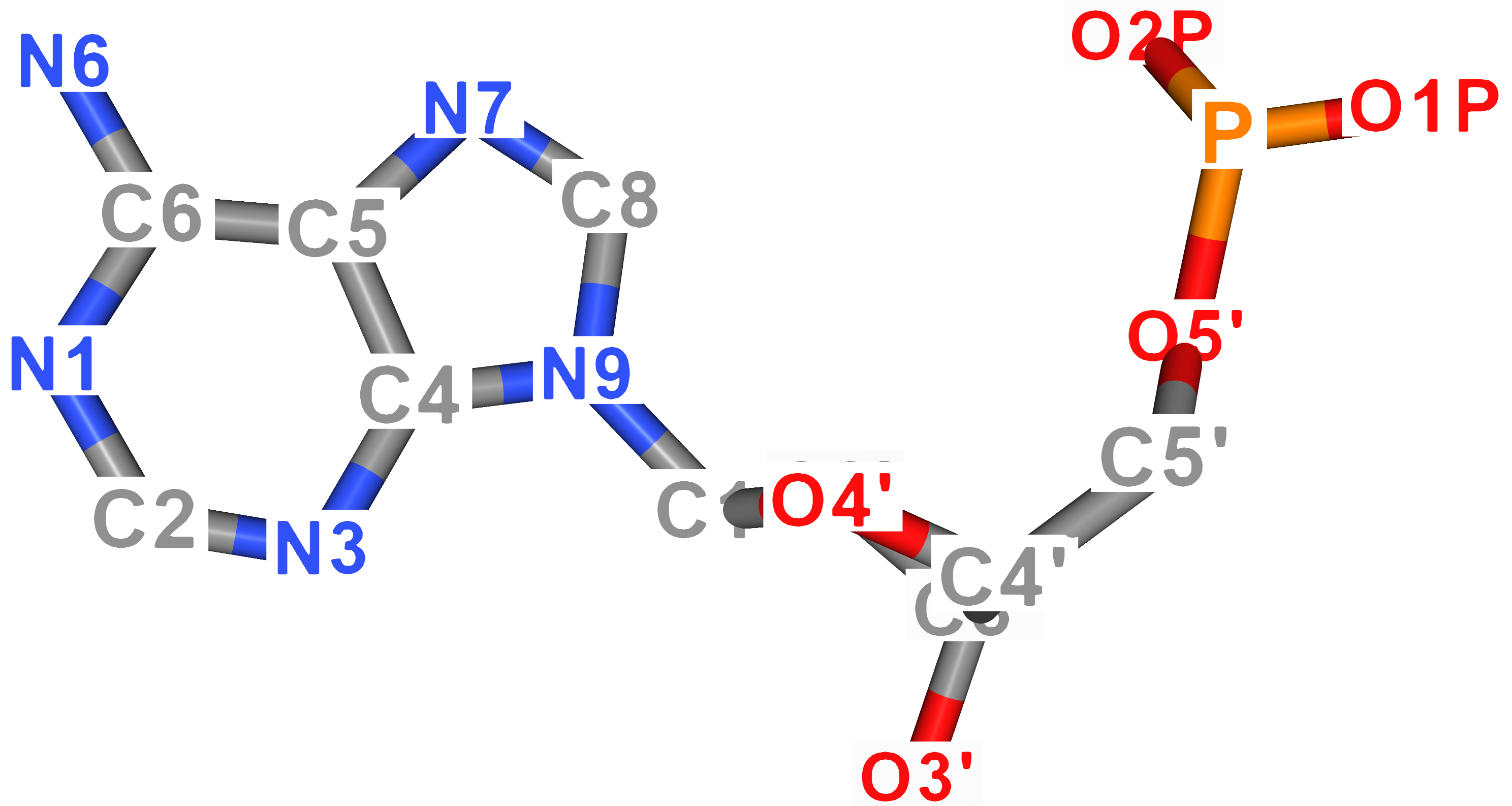

Supplement: Supplementary file 1 [file molecules-27-00063-s001.zip › unf-1.0.0_finalized/docs/nucl_da.png]

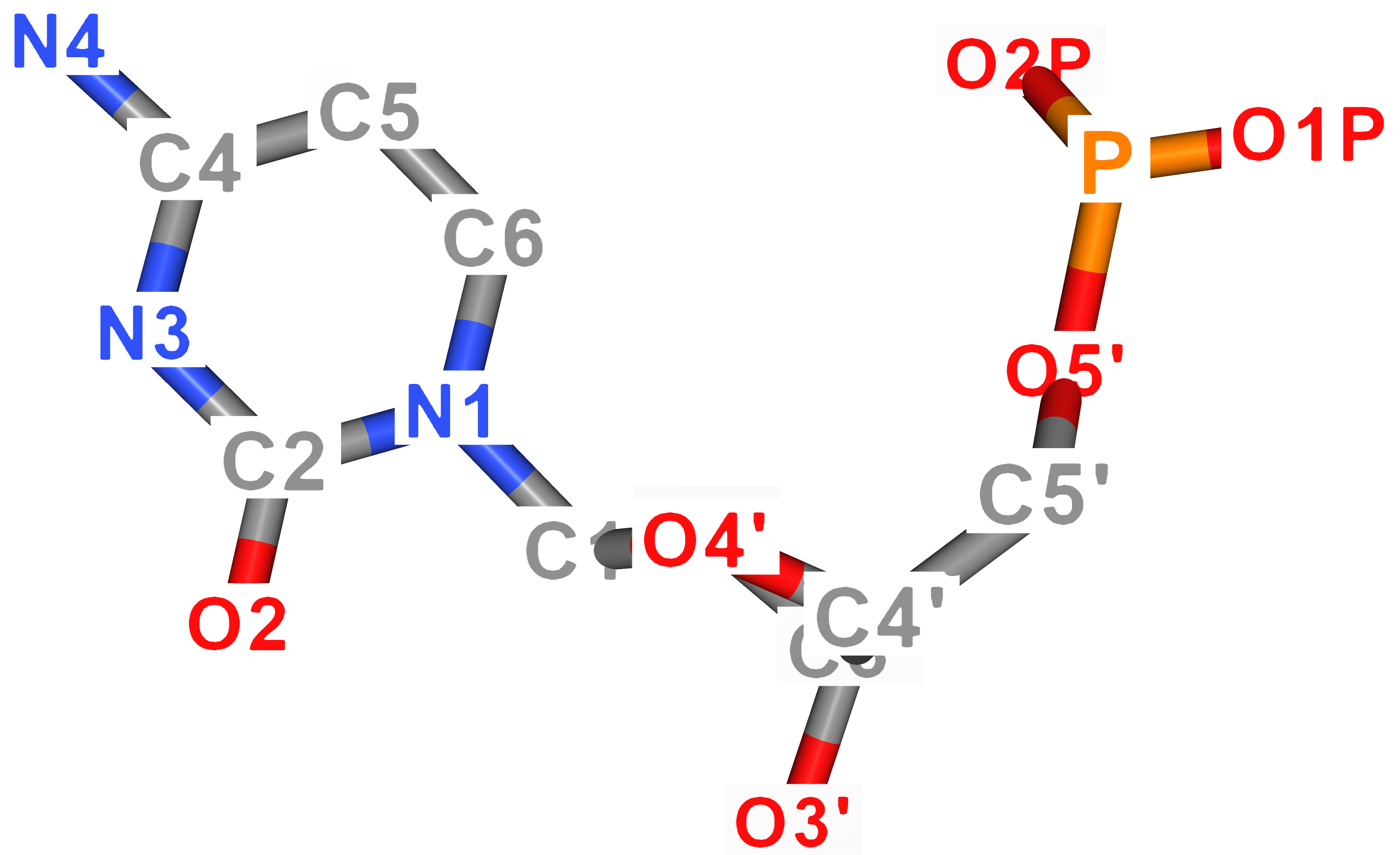

Supplement: Supplementary file 1 [file molecules-27-00063-s001.zip › unf-1.0.0_finalized/docs/nucl_dc.png]

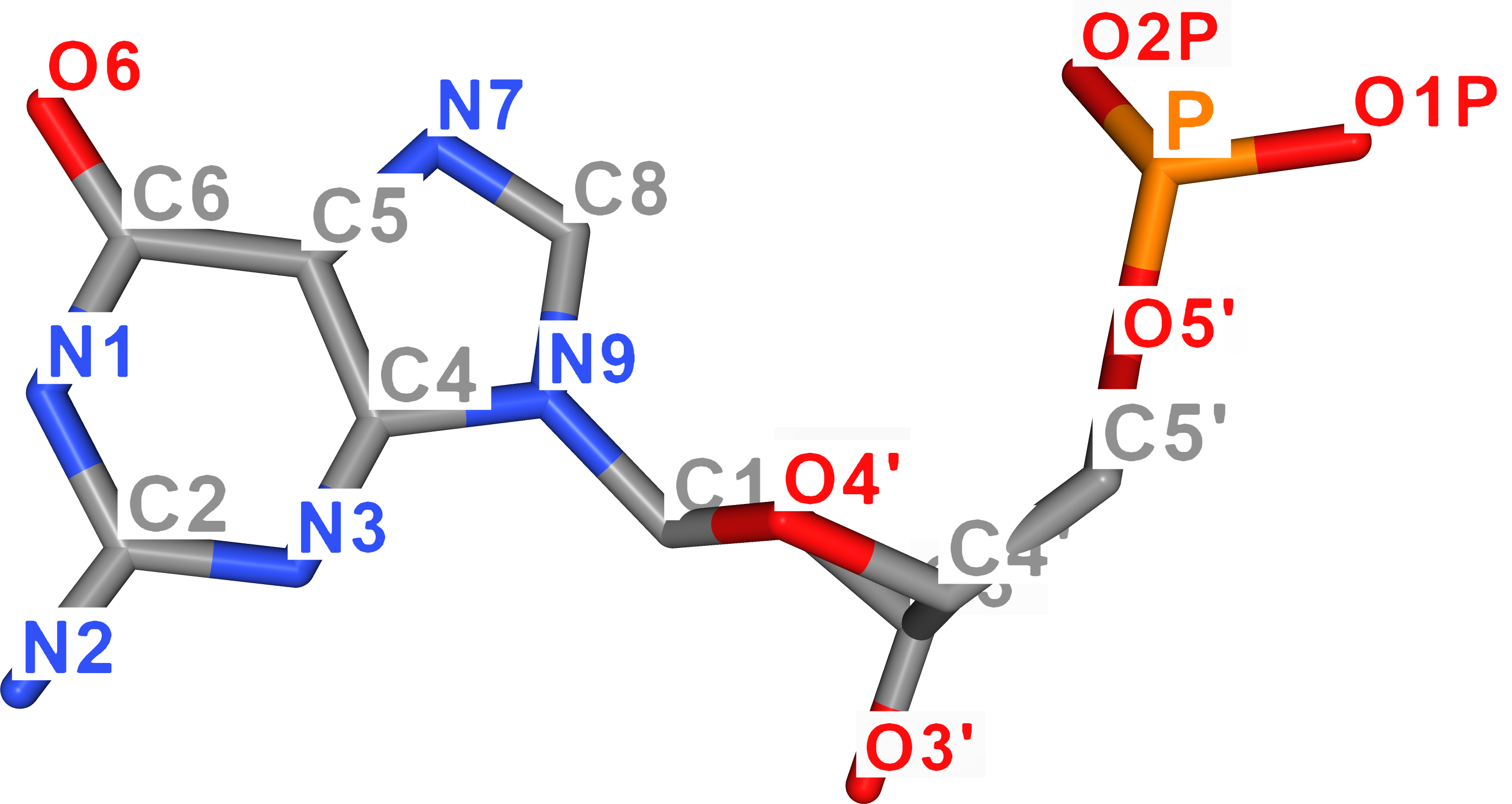

Supplement: Supplementary file 1 [file molecules-27-00063-s001.zip › unf-1.0.0_finalized/docs/nucl_dg.png]

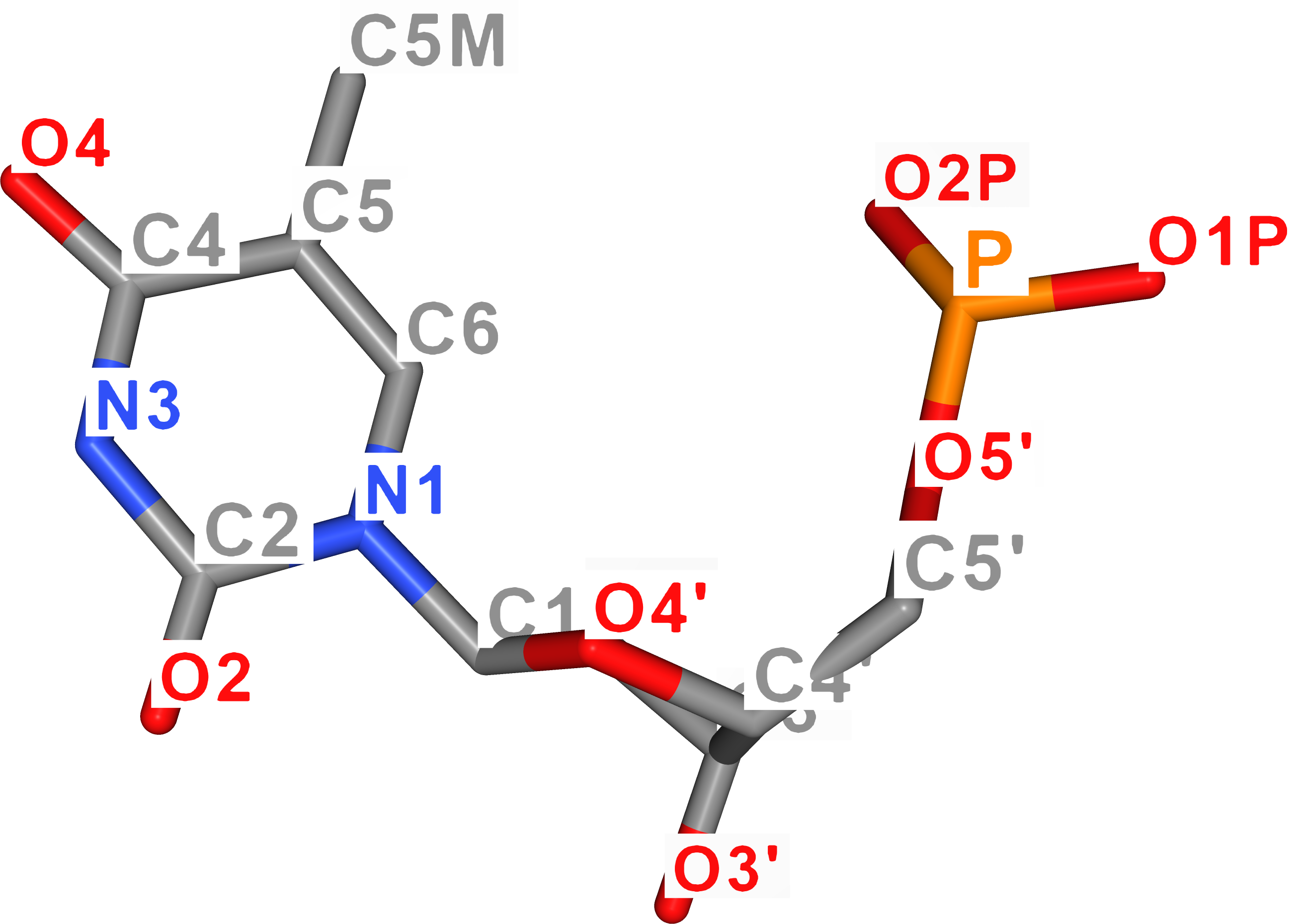

Supplement: Supplementary file 1 [file molecules-27-00063-s001.zip › unf-1.0.0_finalized/docs/nucl_dt.png]
